# RNA-Seq transcriptome reveals different molecular responses during human and mouse oocyte maturation and fertilization

**DOI:** 10.1186/s12864-020-06885-4

**Published:** 2020-07-10

**Authors:** Zheng-Hui Zhao, Tie-Gang Meng, Ang Li, Heide Schatten, Zhen-Bo Wang, Qing-Yuan Sun

**Affiliations:** 1 State Key Laboratory of Stem Cell and Reproductive Biology, Institute of Zoology, Chinese Academy of Sciences, Beijing, China; 2 University of Chinese Academy of Sciences, Beijing, China; 3 Fertility Preservation Lab, Reproductive Medicine Center, Guangdong Second Provincial General Hospital, Guangzhou, 510317 China; 4 Department of Veterinary Pathobiology, University of Missouri, Columbia, MO 65211 USA

**Keywords:** Oocyte maturation, Transcriptome, Fertilization, Transcripts degradation

## Abstract

**Background:**

Female infertility is a worldwide concern and the etiology of infertility has not been thoroughly demonstrated. Although the mouse is a good model system to perform functional studies, the differences between mouse and human also need to be considered. The objective of this study is to elucidate the different molecular mechanisms underlying oocyte maturation and fertilization between human and mouse.

**Results:**

A comparative transcriptome analysis was performed to identify the differentially expressed genes and associated biological processes between human and mouse oocytes. In total, 8513 common genes, as well as 15,165 and 6126 uniquely expressed genes were detected in human and mouse MII oocytes, respectively. Additionally, the ratios of non-homologous genes in human and mouse MII oocytes were 37 and 8%, respectively. Functional categorization analysis of the human MII non-homologous genes revealed that cAMP-mediated signaling, sister chromatid cohesin, and cell recognition were the major enriched biological processes. Interestingly, we couldn’t detect any GO categories in mouse non-homologous genes.

**Conclusions:**

This study demonstrates that human and mouse oocytes exhibit significant differences in gene expression profiles during oocyte maturation, which probably deciphers the differential molecular responses to oocyte maturation and fertilization. The significant differences between human and mouse oocytes limit the generalizations from mouse to human oocyte maturation. Knowledge about the limitations of animal models is crucial when exploring a complex process such as human oocyte maturation and fertilization.

## Background

Ovarian folliculogenesis is an extremely species-specific process and the formation of a mature oocyte starting from a primordial follicle is completed in several weeks in mice [1], but several months in human [2]. Although certain molecular mechanisms underlying fundamental functions of oocyte maturation should be conserved among gamogenetic species [3], the differences between species in oocyte maturation need to be considered. For instance, protein synthesis is essential for germinal vesicle breakdown (GVBD) in human [4] but not in mice [5]. Similarly, increased level of cyclic adenosine monophosphate (cAMP) or cyclic guanosine monophosphate (cGMP) promotes the GVBD in nemertean oocytes, but instead significantly blocks the GVBD in mammals [6]. In addition, physiological concentrations of glucorticoids do not affect mouse oocyte maturation, but typically inhibit the nuclear maturation of pig oocytes [7]. The non-homologous genes that are different among species may contribute to these species-specific molecular pathways.

Fusion of sperm and oocyte is a common aspect that initiates embryo development in gamogenetic species. The transition from oocyte to embryo mainly relies on maternal RNAs and proteins that are generated during oocyte growth [8]. Fully-grown GV oocytes are transcriptionally silent before meiosis resumption until zygote genome activation occurs after fertilization [9–11]. The necessary transcripts deposited in fully-grown GV oocytes are produced during the period of oocyte growth, which is essential for oocyte maturation and fertilization [12]. Also, the selective degradation of transcripts that occurs during oocyte maturation is required for meiotic maturation and oocyte-to-embryo transition [13–15]. Although human and mouse oocytes undergo degradation of maternal mRNAs during oocyte maturation, the time period of oocyte to embryo transition is different between human and mouse.

Several studies have compared the microarray data [16–18], however the differences in oocyte maturation and fertilization between human and mouse have not been fully characterized. Here, we compared the RNA-Seq data of human and mouse oocytes during the transition from the GV stage to the MII stage. As a result, 2243 and 2488 transcripts are completely degraded during oocyte maturation in mouse and human, respectively. Compared to the GV oocytes, 430 and 3790 transcripts appear exclusively in mouse and human MII oocytes, respectively. Moreover, the ratio of non-homologous genes is significantly different between human and mouse oocytes. Collectively, these data suggest that the shared and exclusively expressed transcripts would discriminate between common and species-specific molecular mechanisms that regulate oocyte maturation and fertilization.

## Results

### Slight differences in molecular features between mouse GV and MII oocytes

**Fig. 1:**
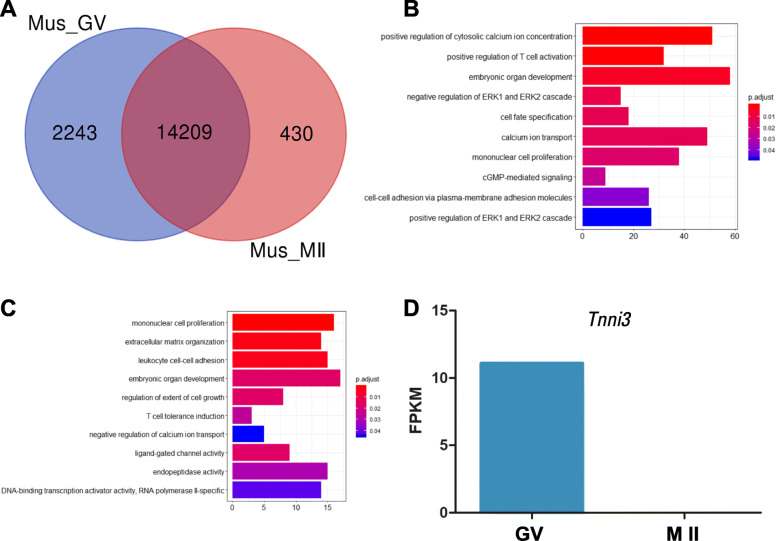
The difference in gene expression patterns between mouse GV and MII oocytes. **a**: Overlap of differentially expressed genes identified between GV and MII oocyte comparisons. **b**: The significant biological processes within differentially expressed genes unique to the GV oocytes. **c**: The enriched GO categories of differentially expressed genes in human MII oocytes. **d**: The expression level of *Tnni3* in GV and MII oocytes

Differences in gene expression profiles between mouse GV and MII oocytes were analyzed to determine the significantly changed transcripts that may contribute to meiotic maturation and fertilization processes. As expected, several transcripts that enriched in meiosis I cell cycle process degraded strikingly during GV to MII transition (Table 1). In the present study, the number of genes that are uniquely expressed in mouse GV and MII oocytes were 2243 and 430, respectively (Additional files 1 and 2). And 14,209 genes were co-expressed at both GV and MII stages (Fig. 1a). During the GV to MII transition, 2243 transcripts were selectively completely degraded. Of particular note, 430 transcripts appeared to be expressed uniquely in MII oocytes.

**Table 1:** Degradation of transcripts during mouse oocyte GV-to-MII transition

Gene	GV FPKM	MII FPKM
**Meiosis I cell cycle process**		
*Ccdc155*	36.7227	1.45956
*Cntd1*	2.49518	0.967175
*Cep63*	11.3733	1.94341
*Mlh1*	11.4869	0.343089
*Top2b*	2.62691	1.2193
*Cdc25c*	9.49562	0.884645
*Syde1*	3.37878	0.42659
*Psmd13*	2.53468	0.383028
*Ercc1*	12.7466	1.00127
*Topbp1*	7.54961	1.90707
*Eme2*	4.15232	0.297086
**Mitochondrion organization**		
*Coa4*	11.9735	1.01023
*Tomm40*	26.1551	0.763054
*Hsd17b10*	11.0287	0.491451
*Prdx3*	20.9225	1.37221
*Cox16*	27.8022	0.713859
*Mtch2*	2.45931	0.200191
*Mrpl15*	7.85712	0.458739
*Wdr45*	3.63553	0.667861
*Trp73*	5.74592	1.35138
**Cytoplasmic translation**		
*Rpl24*	5.34353	0
*Dph5*	4.02856	0.381827
*Eif4h*	105.217	1.32238
*Mcts2*	2.54326	0
*Rpl30*	43.2177	1.20127
*Rps29*	9.1687	0
**RNA modification**		
*Qtrt1*	7.28196	0.177408
*Alkbh3*	5.09442	0.907235
*Aars2*	4.09517	0.199085
*Lcmt2*	6.43164	0.932506
*Nsun5*	5.13521	1.89229
*Ctu2*	4.34528	0.460726

To ascertain differences in gene expression profiles between GV and MII oocytes, we used Clusterprofiler R package to analyze gene ontology of genes that are exclusively expressed in GV and MII oocytes [19]. As shown in Additional files 3 and 4, of the 335 biological processes (BP), 239 are significantly correlated with the transcripts that are degraded during the GV to MII transition, whereas 96 are closely associated with the transcripts that appeared in MII oocytes. We compared the GO categories between the two groups, and found that several biological functions are similar in these two groups, such as “mononuclear cell proliferation”, “embryonic organ development”, “cell-cell adhesion” and “T cell functions” (Fig. 1b and c). Moreover, most of the biological processes are closely related. For instance, 41 transcripts are enriched both in “leukocyte cell-cell adhesion” and “regulation of T cell activation” (Additional file 3), which suggests that immunity-related factors may play essential roles in cell adhesion. In addition, 9 transcripts exclusively expressed in the GV stage are involved in the cGMP-mediated signaling pathway (Additional file 3), which indicates that these transcripts may maintain meiotic arrest in fully-grown GV oocytes. On the other hand, many genes expressed in the GV oocytes were enriched in calcium ion transport. For example, *Tnni3* is a critical component in the calcium ion regulatory system, which is involved in developmental regulations [20]. Of particular note, the expression level of *Tnni3* decreases significantly during the transition from GV to M II oocytes (Fig. 1d). Moreover, several genes involved in negative regulation of calcium ion transport were enriched in MII oocytes, indicating that the calcium ion transport process is active in fully-grown GV oocytes and that calcium ion homeostasis may be differentially regulated in GV oocytes and MII oocytes. The GO terms indicate that newly appearing genes in MII oocytes may play an important role in oocyte maturation and fertilization.

### Specific gene expression patterns in oocytes before and after fertilization

**Fig. 2:**
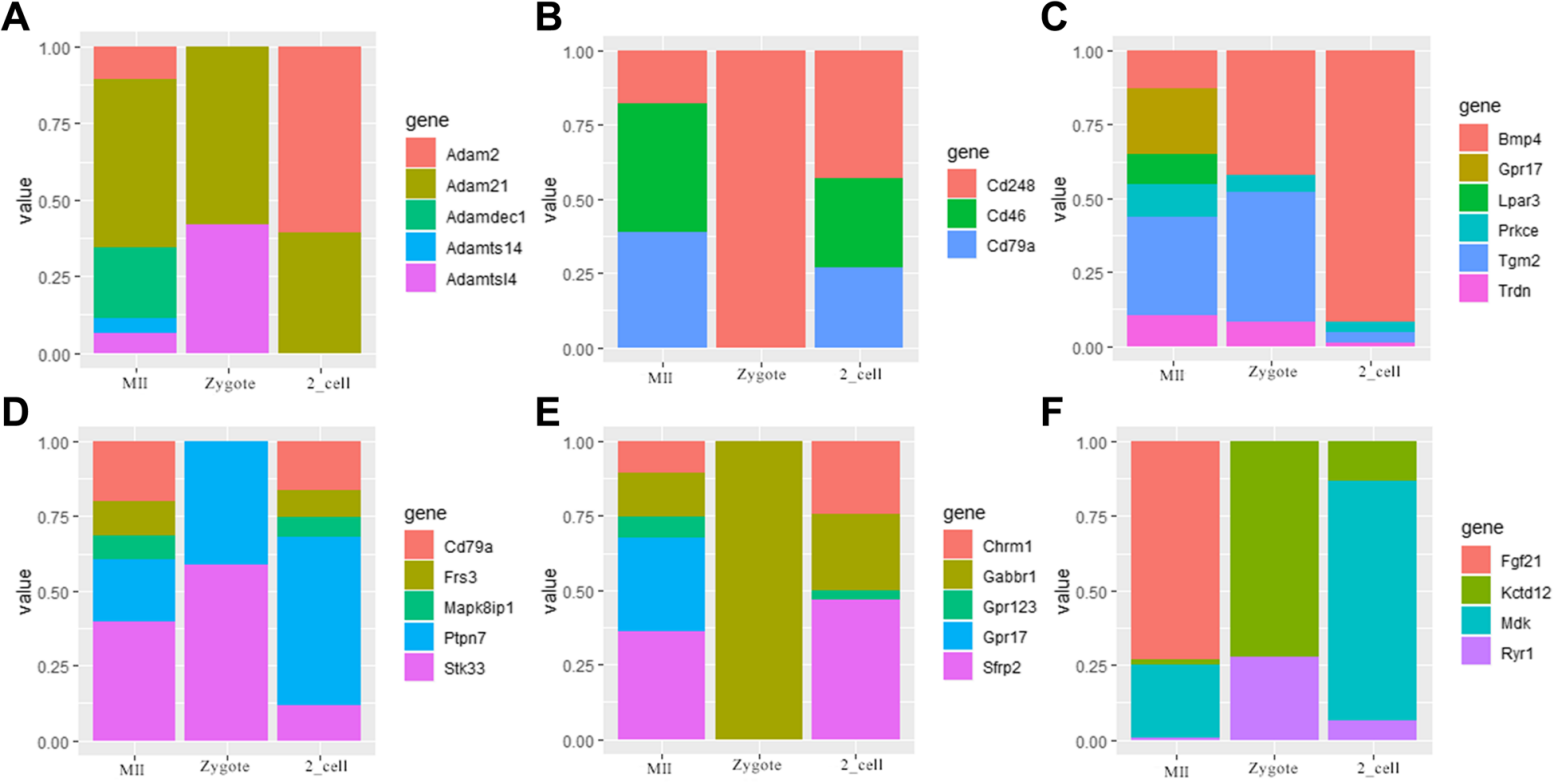
Dynamic changes in gene expression in eggs around fertilization. **a**: Relative expression of Adam gene family. **b**: Relative expression of Cd gene family. **c**: Relative expression of Ca^2+^ homeostasis-related genes. **d**: Relative expression of MAPK signaling pathway-related genes. **e**: Relative expression of G protein-coupled peptide receptor activity-related genes. **f**: Relative expression of cAMP-PKA pathway-related genes

Mammalian fertilization requires recognition, interaction and fusion between sperm and the mature oocyte, which subsequently initiates embryo development through zygote genome activation [21]. To further determine the dynamic changes and roles of genes specifically expressed in MII oocytes, we analyzed the transcriptomes of MII oocytes, zygotes and two-cell embryos (GSE71434) [22]. The ADAM and CD gene families are essential for fertilization [23]. However, we could not detect the expression of *Adam2*, *Cd46* and *Cd79a* in the zygote stage, which indicates that these genes may play roles in the recognition and fusion stage between sperm and the oocyte (Fig. 2a and b). In addition, calcium oscillations are critical during fertilization, which triggers oocyte activation and cell cycle resumption [24]. The expression level of *Bmp4* increases during fertilization (Fig. 2c), which may cooperate with Ca^2+^ to regulate the SMAD1/5 signaling pathway [25]. Moreover, several signaling pathways are critical for fertilization; the *Ptpn7* and *Stk33* that are enriched in mitogen-activated protein kinase (MAPK) pathway and *Gabbr1* that is involved in G protein-coupled receptor pathway play important roles in the oocyte-to-embryo transition (Fig. 2d and e). In the cAMP-PKA pathway, there is a decreased expression of *Fgf21* and increased expression of *Kctd12* during fertilization (Fig. 2f). Collectively, these data suggest that the genes that are exclusively expressed in MII oocytes may play important roles in fertilization.

### Significant differences in molecular features between human GV and MII oocytes

**Fig. 3:**
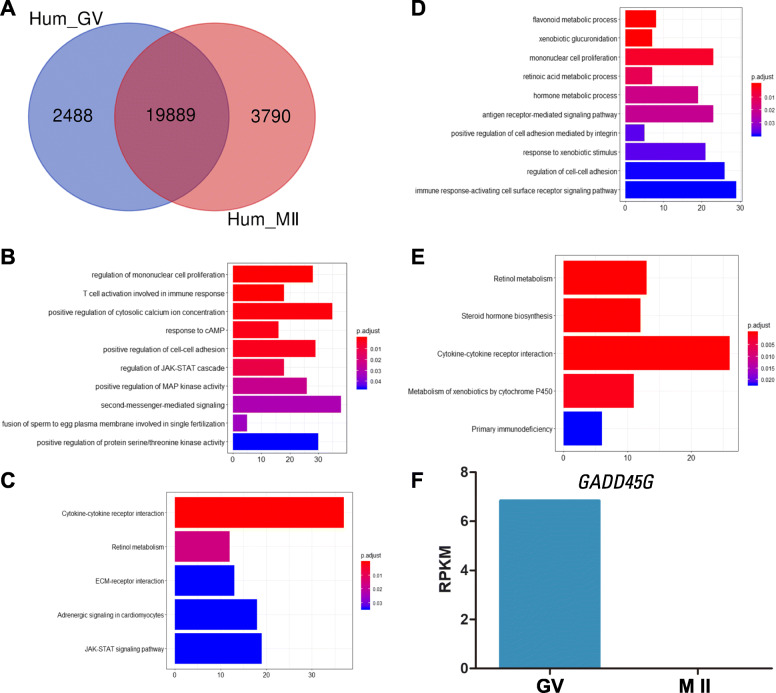
The different gene expression patterns in human GV and MII oocytes. **a**: Overlap of differentially expressed genes between human GV and MII oocytes. **b**: The enriched biological processes within differentially expressed genes unique to the human GV oocytes. **c**: The enriched signaling pathways of differentially expressed genes in human GV oocytes. **d**: The enriched GO terms within differentially expressed genes unique to the human MII oocytes. **e**: The enriched signaling pathways of differentially expressed genes in human MII oocytes. **f**: The expression level of *GADD45G* in GV and MII oocytes

To explore the molecular mechanisms in human oocyte maturation, we compared the gene expression profiles between human GV and MII oocytes. The numbers of genes that were exclusively expressed in human GV and MII oocytes were 2488 and 3790, respectively (Additional files 5 and 6). A total of 19,889 genes were co-expressed in both GV and MII stages (Fig. 3a). During the GV-to-MII transition, 2488 transcripts were selectively completely degraded. Additionally, the transcripts that enriched in mitochondrial translational termination and cytosolic transport degraded dramatically in the process of oocyte maturation (Table 2). In contrast to mouse MII oocytes, there were 3790 transcripts exclusively expressed in MII oocytes.

**Table 2:** Degradation of transcripts during human oocyte GV-to-MII transition

Gene	GV RPKM	MII RPKM
**Translational termination**		
*MRPL28*	10.3237999	0.930954689
*MRPS11*	4.514520788	0.540265841
*UPF1*	2.066086591	1.165330501
*MTRF1*	3.654566571	1.87621318
*MRPS22*	8.353415364	0.713688366
**Cytosolic transport**		
*PIK3C3*	3.50643722	1.239482851
*STX5*	3.076809772	0.299874951
*GOSR1*	3.701789336	1.399290475
*WIPI1*	2.162167036	1.177426334
*ANKRD27*	6.941615019	1.914800745
*YKT6*	4.572012219	0.565371648
*GGA1*	2.039797289	0.258461412
*WDR91*	2.82799633	0.248637966
*SNF8*	3.836114823	1.310913087
*SYS1*	4.181614332	1.120275234
**Golgi vesicle transport**		
*RNF139*	7.365387964	1.444282184
*CCDC22*	5.252229072	0.649054735
*TMED1*	14.80813427	1.352350738
*LMAN2L*	2.639099354	1.064585623
*PREB*	9.939356968	1.762995138
**Vesicle docking**		
*VPS11*	2.261760319	0.278176398
*SNPH*	3.842344389	1.252070021
*VPS33A*	2.495807447	0.291106366
*RAB3D*	2.191925935	1.123575285
*STX10*	9.092859067	1.0749016
*RABEPK*	4.980868195	1.067827433

To further determine the features of human GV and MII oocytes, we performed GO and KEGG analysis of the genes that are uniquely expressed in human GV and MII oocytes. The gene ontology analysis revealed 257 categories, of which 217 are closely associated with the transcripts that are lost during oocyte maturation, whereas 40 are the transcripts that appear in MII oocytes (Additional files 7 and 8). Also, 34 KEGG pathways were detected among stage exclusively expressed transcripts (Additional files 9 and 10). Furthermore, we compared the GO terms and KEGG pathways between the two groups, and there were several similar categories such as “mononuclear cell proliferation”, “cell-cell adhesion”, “Retinol metabolism” and “cytokine-cytokine receptor interaction” (Fig. 3b-e). On the other hand, 16 transcripts that were exclusively expressed in the GV stage were enriched in the category of response to cAMP (Fig. 3b; Additional file 7), which suggests that these transcripts could participate in the regulation of meiotic arrest in fully-grown GV oocytes [26]. In addition, the term of positive regulation of cell adhesion mediated by integrin suggests that genes, such as *CXCL13*, *SKAP1* and *FOXC2*, specifically expressed in MII stage may play an important role in oocyte maturation and fertilization (Fig. 3d; Additional file 8). Additionally, growth arrest and DNA damage 45G (*GADD45G*) is a reproduction related gene that is enriched in MAPK activities [27–29]. The complete degradation of *GADD45G* during the GV-to-MII transition suggests its role in oocyte maturation (Fig. 3f).

### Comparison of gene expression profiles between human and mouse oocytes

**Fig. 4:**
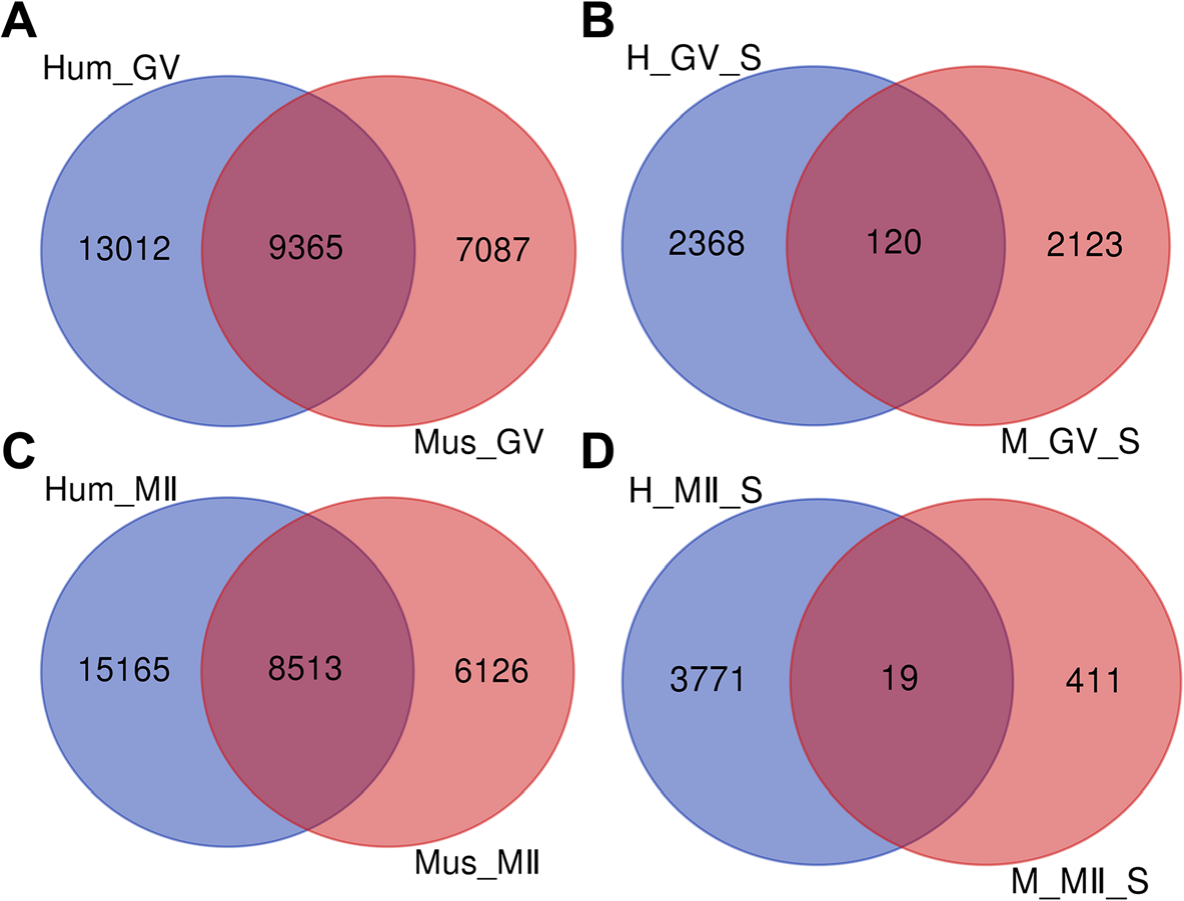
The different gene expression patterns between human and mouse oocytes. **a**: Venn diagram shows overlapping of differentially expressed genes in human and mouse GV oocytes. **b**: Overlap of differentially expressed genes between human GV-specific genes and mouse GV-specific genes. **c**: Venn diagram shows overlapping of differentially expressed genes in human and mouse MII oocytes. **d**: Overlap of differentially expressed genes between human MII-specific genes and mouse MII-specific genes

Oogenesis is a species specialized developmental process and the mouse is not the most suitable animal model for humans, especially when exploring oocyte maturation and fertilization [30, 31]. To examine the differences in regulating oocyte maturation, we compared the gene expression profiles between human and mouse oocytes. A total of 9365 genes overlapped between human and mouse GV oocytes (Fig. 4a), of which only 120 transcripts specifically expressed in the GV oocytes overlapped in human and mouse (Fig. 4b). Furthermore, 8513 genes overlapped between human MII and mouse MII oocytes (Fig. 4c), of which only 19 transcripts that specially were expressed in MII oocytes overlapped in human and mouse (Fig. 4d). Collectively, these data suggest that there is a different regulatory network responsible for human and mouse oocyte maturation.

### The ratio of non-homologous genes in human and mouse MII oocytes

**Fig. 5:**
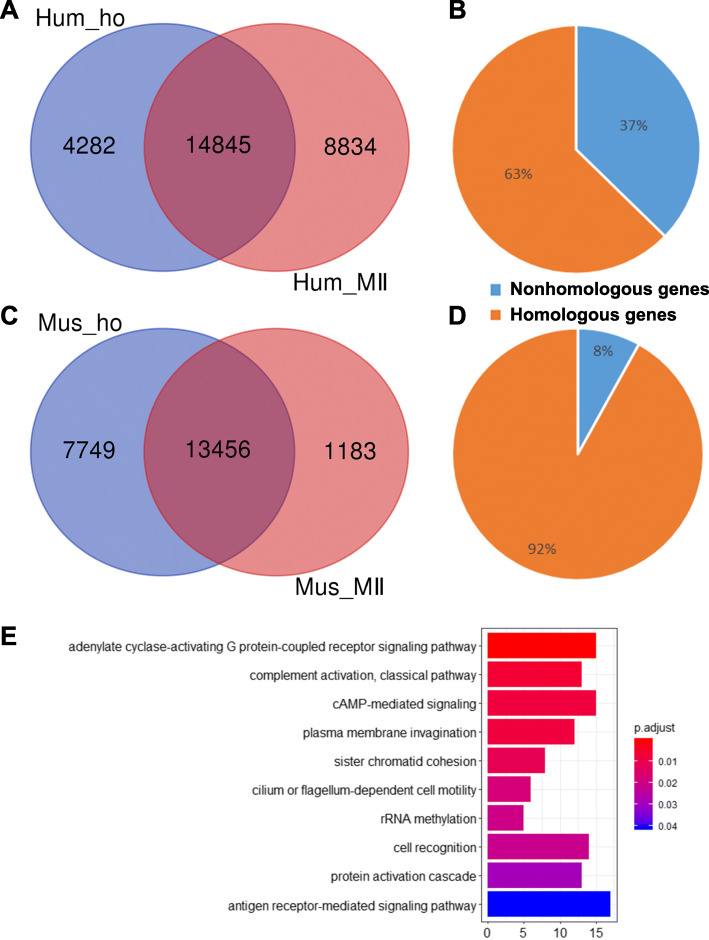
The difference of non-homologous genes between human and mouse MII oocytes. **a**: Venn diagram shows overlapping genes in human homologous genes database and human MII expressed genes. **b**: The ratio of non-homologous genes in human MII oocytes. **c**: Venn diagram shows overlapping genes in mouse homologous genes database and mouse MII expressed genes. **d**: The ratio of non-homologous genes in mouse MII oocytes. **e**: The enriched biological processes within non-homologous genes unique to the human MII oocytes

The ratio of non-homologous genes in oocytes could pinpoint the differences among species. Exclusively expressed genes would discriminate the species-specific molecular pathways between human and mouse oocyte maturation. To further determine the ratio of non-homologous genes in human and mouse MII oocytes, we downloaded the homologous gene database from NCBI (ftp://ftp.ncbi.nih.gov/pub/HomoloGene/). In human MII oocytes, 8834 genes are non-homologous genes, and the ratio of non-homologous genes is 37% (Fig. 5a and b; Additional file 11). Whereas 1183 genes are non-homologous genes in mouse MII oocytes, and the ratio of non-homologous genes is 8% (Fig. 5c and d; Additional file 12).

To further explore the functions of non-homologous genes, we performed GO analysis on non-homologous genes in human and mouse MII oocytes. The enriched biological functions of human non-homologous genes are related to fertilization, such as “plasma membrane invagination”, “sister chromatid cohesion” and “cell recognition” (Fig. 5e; Additional file 13). Interestingly, the “cAMP-mediated signaling” and “adenylate cyclase-activating G protein-coupled receptor signaling pathway” are also enriched in human MII oocytes (Additional file 13). In contrast to the “response to cAMP” in human GV oocytes, all transcripts enriched in these two categories are Adhesion G Protein-Coupled Receptor (ADGR) gene families (Additional file 13), which may be involved in the regulation of calcium ion homeostasis in human MII oocytes [32]. However, we did not acquire the GO terms of non-homologous genes in mouse MII oocytes (data not shown). These results indicate that there is a significant difference between human and mouse MII oocytes.

## Discussion

Female infertility and related reproductive disorders have overall health implications. The etiology of infertility remains elusive, and the exploration of etiology is predominantly based on studies of animal models. However, oogenesis is a species specialized process [31]. Although mouse is suited for studies of oocyte maturation and fertilization, the differences between mouse and human also need to be focused. Comparative analysis of RNA-Seq data has provided informative insights into the differences between human and mouse oocytes.

Oocyte maturation and fertilization have been explored for many years [21, 33, 34]; however, little is known regarding the different mechanisms between human and mouse. Here, we analyzed the transcriptomes of human and mouse GV and MII oocytes to explore the differences between human and mouse oocytes at the transcriptional level [22, 35]. As reported above, the regulation of oocyte maturation and fertilization exhibits different features in human and mouse oocytes. For example, compared to GV oocytes, the number of apparent transcripts in mouse and human MII oocytes are 430 and 3790, respectively (Figs. 1a and 3a). Noticeably, the number of transcripts may be slightly biased due to the limited number of samples studied and the different stages of functional annotation of the genomes. Additionally, differential gene expression analysis between human and mouse oocytes identified numerous significant differentially expressed genes that were enriched for typical or species-specific pathways and biological processes. The transcriptomes of human and mouse oocytes are highly variable, and the human oocytes exhibit more complexity than mouse oocytes.

Extensive degradation of transcripts occurs during oocyte maturation, which is required for oocyte to embryo transition. Several studies have explored the mechanisms of maternal mRNA decay in mouse oocytes. For example, *BTG4*, *CNOT6L* and *ZAR1/2* participate in the destruction of target specific transcripts during oocyte maturation [13, 34, 36]. However, the differences of selectively degraded transcripts between human and mouse have not been explored in detail. In this study, we have presented results showing that the number of completely degraded transcripts is similar (2243 and 2488, respectively), but only 120 transcripts were overlapped. As expected, the majority of GO categories of these degraded transcripts between mouse and human oocytes are different. Therefore, the difference in selectively degraded transcripts between human and mouse oocytes suggests deviations of regulatory mechanisms that control oocyte maturation.

On the other hand, there are certain transcripts that appear to be up-regulated in MII oocytes. Moreover, the majority of GO terms of these transcripts between mouse and human oocytes are different except for some immunity-related categories. Although we didn’t perform extra experiments to validate the results with human oocytes, several studies have reached some similar results, especially in the enriched signaling pathways at the corresponding stages [37–39]. As we all know, fully-grown GV oocytes are transcriptionally silent before resumption of meiosis until after fertilization when zygote genome activation occurs [8]. Therefore, the transcripts that exclusively appeared in MII oocytes may not be newly transcribed. And the reason of origin of these transcripts needs to be further explored.

The novelty and conservation of gene expression profiles shape the differences of species [3]. Two-level analysis of transcriptome demonstrated that the significant differences in molecular responses exist between human and mouse oocytes. The ratio of non-homologous genes in human MII oocytes is four times higher than that in mouse MII oocytes. However, most GO categories of non-homologous genes in human MII oocytes are conserved biological processes including “adenylate cyclase-activating G protein-coupled receptor signaling pathway” and “cAMP-mediated signaling”. Unexpectedly, we hardly detected any GO terms in non-homologous genes of mouse MII oocytes. Therefore, the strong evidence for diversification of non-homologous genes prompts us to hypothesize that the functions of novel loci expressed in the oocytes are shaped by the forces of gametic selection that target the processes unique to individual species. Therefore, it shows potential shortcomings related to the use of mouse model to explore the oocyte maturation process in human.

## Conclusions

In summary, human and mouse oocytes exhibit divergent transcriptomes at the fully-grown GV and MII stages, probably deciphering the differential molecular response to oocyte maturation and fertilization. Critical factors involved in oocyte maturation were found to be differentially expressed between human and mouse oocytes. Moreover, human MII oocytes exhibited a higher ratio of non-homologous genes compared to mouse MII oocytes, which were enriched for various biological processes that play important roles during oocyte maturation. These findings show significant differences in gene expression profiles between human and mouse oocytes, limiting the generalizations from mouse to human oocyte maturation. Knowledge about the limitations of animal models is crucial when exploring a complex process such as human oocyte maturation and fertilization.

## Methods

### Comparation of library preparation and data processing

Mouse (C57BL/6 N) oocyte and early embryo transcriptome data were obtained from Zhang et al. [22], who used Smart-Seq2 protocol with slightly modification to prepare the RNA-Seq libraries [40]. All cDNA libraries were fragmented and then sequenced on Illumina HiSeq platform. After sequencing, the raw reads in the fastq format were first processed and the clean reads were obtained by removing adapter sequences and low-quality reads from raw data. Next, the clean reads were aligned to the reference genome (mm9) using TopHat (version 2.0.11). Based on the comparison results, the sequencing data were assembled by Cufflinks (version 2.0.2). And the normalization of the gene expression values was based on the fragments per kilobase of exon per million reads mapped (FPKM).

Human oocyte transcriptome data were obtained from Reyes et al. [35]. The total RNA of GV and MII human oocytes were purified according to the manufactures’ protocol, which were then amplified using SMARTer Ultra Low Input RNA HV kit (Clontech, USA). The cDNA libraries were fragmented and then sequenced on a paired-end 2 × 100 bp Illumina Hiseq 2500 platform. After sequencing, the raw reads were processed using CLC Genomics Workbench (CLC, v7.5.1; Qiagen, USA) to remove the Clontech IS PCR primer (5′- AAGCAGTGGTATCAACGCAGAGTAC-3′) from the raw data. The clean reads were then aligned to the annotated human genome (GRCh38.82) according to the RNA-Seq tool instruction with small modification. CLC normalized expression values for each gene as total exon reads per kilobase of transcript per million mapped reads (RPKM). Differential expression analysis was performed using the DESeq2 package (v1.10.1) through mean normalized exon reads that exported from CLC Genomics Workbench.

Different library preparation and data processing procedures could lead to the bias of detected genes and gene expression levels. To reduce the bias of data, we selected all the transcripts, whose expression levels > 0 in this study.

### Expression analysis of transcriptomes

Normalized FPKM and RPKM values for mouse and human oocyte maturation and fertilization stages were obtained from GEO database (GSE71434_FPKM_stage.txt.gz; GSE95477_Differentially_Expressed_Genes_and_RPKM_Values.csv.gz). Data from the following stages were used in this study: 8-week GV oocytes, MII oocytes, zygotes and 2-cell embryos from Bing-Jie Zhang et al. [22] and young women (< 30 years) GV and MII oocytes from Reyes et al. [35]. The transcripts with average FPKM or RPKM > 0 at all above stages were retained for further analysis. To identify the genes uniquely expressed in a given group, we compared the two groups of samples through Draw Venn Diagram (http://bioinformatics.psb.ugent.be/webtools/Venn/). In addition, homologous gene database ‘build68’ was downloaded (ftp://ftp.ncbi.nih.gov/pub/HomoloGene/) to calculate the ratios of the non-homologous genes in mouse and human MII oocytes.

### Gene ontology and KEGG analysis

Gene ontology and KEGG analysis was performed by using the ClusterProfiler R package [19]. Given a list of genes, symbol gene IDs were translated into entre IDs through bitr function. The GO and KEGG analysis were conducted through enrich GO and enrich KEGG functions, respectively. The analysis of the enrichment of uniquely expressed genes was conducted and a corrected *P*-value≤0.05 was considered to indicate significant gene enrichment.

## Supplementary information

**Additional file 1.** Mouse GV oocyte-specific expressed gene list.

**Additional file 2.** Mouse MII oocyte-specific expressed gene list.

**Additional file 3.** GO categories of mouse GV oocyte-specific expressed genes.

**Additional file 4.** GO categories of mouse MII oocyte-specific expressed genes.

**Additional file 5.** Human GV oocyte-specific expressed gene list.

**Additional file 6.** Human MII oocyte-specific expressed gene list.

**Additional file 7.** GO categories of human GV oocyte-specific expressed genes.

**Additional file 8.** KEGG categories of human GV oocyte-specific expressed genes.

**Additional file 9.** GO categories of human MII oocyte-specific expressed genes.

**Additional file 10.** KEGG categories of human MII oocyte-specific expressed genes.

**Additional file 11.** Human MII oocyte non-homologous gene list.

**Additional file 12.** Mouse MII oocyte non-homologous gene list.

**Additional file 13.** GO categories of human MII oocyte non-homologous genes.

## Data Availability

The data sets analyzed here are publicly available. The mouse oocyte data set is available on the Gene Expression Omnibus (GEO) database, and the accession number is GSE71434, and the human oocyte data set is available on the GEO database, and the accession number is GSE95477. The reference genome (mm9) was downloaded from the website (http://hgdownload.cse.ucsc.edu/goldenPath/mm9/chromosomes/). The annotated human genome (GRCh38.82) was downloaded from the website (https://www.ncbi.nlm.nih.gov/genome/guide/human/).

## Abbreviations

BP: Biological processes
FPKM: Fragments per kilobase of exon per million reads mapped
GADD45G: Growth arrest and DNA damage 45G
GEO: Gene expression omnibus
GO: Gene ontology
IVF: In vitro fertilization
KEGG: Kyoto encyclopedia of genes and genomes
MAPK: Mitogen-activated protein kinase
RPKM: Reads per kilobase of exon per million reads mapped
